# Health-Related Quality of Life in Patients with Primary Adrenal Insufficiency

**DOI:** 10.3390/jcm12237237

**Published:** 2023-11-22

**Authors:** Aleksandra Zdrojowy-Wełna, Alicja Stańska, Jowita Halupczok-Żyła, Dorota Szcześniak, Marek Bolanowski

**Affiliations:** 1 Department of Endocrinology, Diabetes and Isotope Therapy, Wroclaw Medical University, 50-367 Wroclaw, Poland; alicja_kowalewska12@hotmail.com (A.S.); jowita.halupczok-zyla@umw.edu.pl (J.H.-Ż.); marek.bolanowski@umed.wroc.pl (M.B.); 2 Department of Psychiatry, Wroclaw Medical University, 50-367 Wroclaw, Poland; dorota.szczesniak@umed.wroc.pl

**Keywords:** Addison’s disease, PAI, health-related quality of life, HRQoL, primary adrenal insufficiency, AddiQoL

## Abstract

(1) Background: Patients with primary adrenal insufficiency (PAI) suffer from a reduced quality of life. However, clinical factors associated with this impairment remain unclear. The aim of this study was to assess the health-related quality of life (HRQoL) and to evaluate the associations with clinical and hormonal parameters in a group of patients with PAI. (2) Methods: The study included 32 patients with autoimmune PAI, who answered the quality of life in Addison’s disease questionnaire (AddiQoL). Clinical data and hormonal measurements were collected from the patients. (3) Results: The total AddiQoL score of males was significantly higher than that of females (*p* = 0.011). Furthermore, males reached significantly higher scores in each of the four subscales (fatigue—*p* = 0.013, emotional sphere—*p* = 0.048, adrenal insufficiency symptoms—*p* = 0.039, and miscellaneous questions—*p* = 0.034). There was a negative correlation between HRQoL and gonadotropin levels (FSH and fatigue r = (−)0.38, *p* = 0.032; FSH and emotional sphere r = (−)0.416, *p* = 0.018). This study found no significant associations between AddiQoL scores and the presence of autoimmune comorbidities; only fatigue scores were worse in the presence of autoimmune thyroiditis (*p* = 0.034). The doses of hydrocortisone and fludrocortisone in the replacement therapy were not associated with AddiQoL scores. AddiQoL scores correlated negatively with the age of diagnosis (*p* = 0.015). (4) Conclusions: Female sex, higher gonadotropins level, and older age at diagnosis were associated with impaired HRQoL in the studied group of patients with PAI.

## 1. Introduction

Primary adrenal insufficiency (PAI) is a rare chronic disease resulting from the inability of the adrenal glands to produce sufficient amounts of steroid hormones. The reported prevalence in most European countries is about 10 per 100,000 [1]. It was primarily recognized by Thomas Addison and therefore named Addison’s disease. The most common pathophysiological cause of Addison’s disease in European countries is the autoimmune destruction of adrenal glands, with the immune system targeting 21-hydroxylase. Most individuals are diagnosed between 20 and 50 years of age, with a slight female preponderance. About 60% of the patients have more than one autoimmune disease, known as autoimmune polyendocrinopathy, contributing to autoimmune destruction [2]. Patients with PAI need lifelong hormonal replacement therapy with glucocorticoids, mineralocorticoids, and sometimes adrenal androgens. The discontinuation of therapy may cause a life-threatening adrenal crisis, especially during stress, when the dosage of glucocorticoids should be increased. As a result of chronic disease burden and imperfect treatment, patients suffer from increased mortality, worse healthcare outcomes, and reduced quality of life (QoL) [3,4].

The definition of QoL, according to the World Health Organization, includes all aspects of human life, including health, physical and social functioning, income, values, culture, spirituality, and life satisfaction. Health-related quality of life (HRQoL) is a narrower term, focusing on the effects of disease and treatment on QoL. Therefore, HRQoL cannot be compared with that of the general population but gives the possibility of a more personalized approach to the patient. A crucial principle in evaluating QoL is the patient’s perspective. Standardized tools are needed to inform the clinician about the patient’s QoL, otherwise, problems might go unrecognized. Questionnaires like Short-Form 36 (SF-36) or Checklist Individual Strength were used in patients with PAI, showing impaired QoL in comparison to that of healthy controls in spite of treatment [3,5,6]. The most affected categories were vitality, general health perception, emotional role, and increased fatigue [7,8]. In a recent study by Didriksen et al., 494 patients with PAI reported impairment of QoL in comparison to that of controls with the use of the RAND-36 (version of SF-36) questionnaire, especially in perception of physical role in general. The study also revealed gender-specific differences in QoL. The scores for social functioning, vitality, and physical role were mostly affected in males, while females presented the largest impairment of physical role, followed by a reduction of social functioning, vitality, physical function, general health, mental health, and emotional role [9].

The disease-specific quality of life questionnaire in Addison’s disease (AddiQoL) was designed in 2010 by Lovas et al. and included 36 items [10]. After validation it was reduced to 30 questions [11]. It was designed as a more sensitive tool for the evaluation of specific problems in PAI in comparison to generic questionnaires. AddiQoL has shown its benefit as a useful tool to assess the HRQoL of patients with PAI during different therapeutic interventions; however, the number of studies with its use is still small [12,13,14,15]. Interestingly, it has also been reported that the evaluation of AddiQoL may help identify individuals at a higher risk of an adrenal crisis or patients in pre-crisis, because they report lower scores in AddiQoL. It seems crucial as it potentially allows for early diagnosis and treatment, therefore decreasing morbidity and mortality [16].

The predictors and associated factors of HRQoL in adrenal insufficiency are still unclear; however, HRQoL remains impaired irrespective of etiology [5]. Some studies suggest that HRQoL in patients with PAI is worse in females, older patients, patients with a longer latency period between first symptoms and diagnosis, and the existence of other autoimmune diseases and non-autoimmune etiology [9,17]. Other studies have shown impairment of HRQoL irrespective of age, sex, and concomitant diseases. Most available studies also focused on the type of glucocorticoid replacement strategy as a potentially modifiable factor that may influence HRQoL, but the data are inconsistent [8,12,14,18,19]. Additionally, the supplementation of adrenal androgens and its influence on HRQoL remains controversial [6,7,20,21]. These inconsistencies may be partially explained by different methods of HRQoL assessment.

Summarizing, there is a research gap in terms of the predictors of HRQoL in Addison’s disease. Therefore, the objectives of this study were to assess AddiQoL scores in a homogenous group of Polish patients with autoimmune PAI and to evaluate the associations with clinical and hormonal parameters in a prospective observational study.

## 2. Materials and Methods

**Figure 1:**
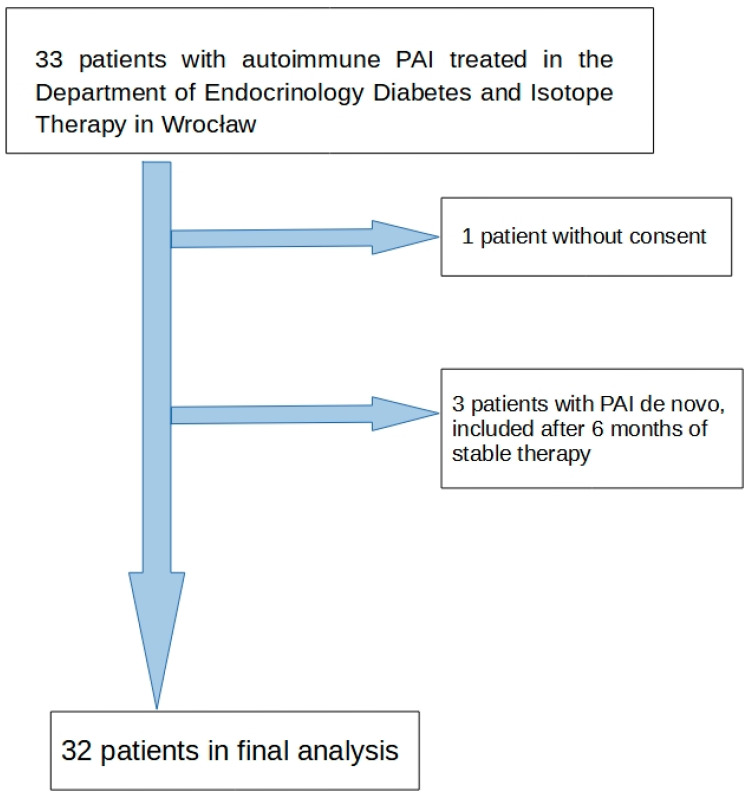
Study flow chart.

The study was designed as prospective and observational with assumed time of recruitment limited to two years. Therefore, all patients with a diagnosis of autoimmune PAI who were followed up in the Department of Endocrinology, Diabetes, and Isotope Therapy, Wrocław Medical University Hospital, between February 2020 and May 2022 were asked to take part in the study. The inclusion criteria were the diagnosis of autoimmune PAI confirmed at least 6 months before inclusion in the study and stable doses of hormonal replacement therapy (hydrocortisone, fludrocortisone, L-thyroxine, and insulin doses were unchanged for at least 6 months; in cases where infections occurred, the doses of hydrocortisone were increased for a short period). Only one patient during this period refused to take part in the study and was not included. During the study duration, three PAI patients in our department were diagnosed de novo, therefore, we waited 6 months to achieve the stabilization of the disease course and treatment before inclusion into the study (Figure 1). The sample size was limited by the duration of the study and the rarity of the disease—32 patients were included.

The diagnosis of PAI was established according to the Endocrine Society Clinical Guidelines on the basis of a corticotropin stimulation test with 250 µg of synthetic ACTH. In case this test was unavailable, a morning cortisol concentration (taken at 7–8.00 a.m.) lower than 5 µg/dL accompanied with an ACTH concentration twofold of the upper reference range and typical PAI symptoms were used alternatively [3]. Hypopituitarism was excluded in all patients. In the case of positive testing for autoantibodies against adrenal 21-hydroxylase in blood (available in 24 subjects) or the coexistence of at least two other autoimmune diseases, with no signs of other causes of adrenal insufficiency, PAI was considered as autoimmune. All patients underwent imaging of the abdomen (ultrasonography or computed tomography) to exclude other causes of PAI.

Each patient was examined by an endocrinologist. The examination included taking a detailed medical history with information about current hormonal replacement therapy and mean doses for the last 6 months, as well as a physical examination including anthropometric measurements and calculation of BMI.

The confounding variables in our study were autoimmune thyroiditis and diabetes as a part of autoimmune polyendocrinopathy. Seventeen females (85%) and four males (33.3%) had autoimmune thyroiditis treated with L-thyroxine supplementation. The TSH levels and L-thyroxine supplementation were stable for at least 6 months before inclusion to the study. The autoimmune diabetes treated with insulin was diagnosed in five females (25%) and one male (8.3%), the doses of insulin were stable and there were no episodes of diabetic ketoacidosis for at least 6 months before the study. A potential design bias is the single-center character of our study.

The following blood laboratory tests were collected (between 7.00 and 8.00 a.m.): dehydroepiandrosterone sulfate (DHEAS), ACTH, follicle-stimulating hormone (FSH), luteinizing hormone (LH), estradiol, total testosterone, thyroid-stimulating hormone (TSH), and free thyroxine (fT4). The concentrations of hormones were measured by the chemiluminescence immunoassay method (Immulite 2000, Siemens Healthcare Diagnostics, Malvern, PA, USA). The reference ranges are presented in Table 1. Urinary free cortisol (UFC) from a 24 h urine collection sample was assessed with the use of a radioimmunoassay method (Immunotech, Beckman Coulter Inc., Prague, Czech Republic). There was no settled reference range in patients receiving hydrocortisone replacement therapy. Espiard et al. have shown that in patients with PAI treated with conventional hydrocortisone replacement therapy, UFC was 3-fold higher than that in controls; *p* < 0.001 (after adjustment for age, sex, and weight) [22].

Each patient completed the validated AddiQoL questionnaire with 30 questions. The validity of the AddiQoL was examined by Øksnes et al. with the use of exploratory factor analysis and Rash analysis, proving good psychometric properties and high reliability [11]. The questions are divided into domains evaluating different aspects: fatigue (questions 1, 2, 3, 4, 5, 23, 26, and 27), emotions (questions 11, 12, 13, 14, 15, 24, 25, and 30), adrenal insufficiency (AI) symptoms (questions 6, 9, 16, 17, 18, 19, 20, 21, and 22) and miscellaneous questions—sleep (questions 7 and 8), sexuality (question 10), impact of concurrent diseases (questions 28 and 29).

Items that indicate a positive impact on QoL were scored from 1 to 6, with reverse scoring for those indicating a negative impact. Scoring was converted in the following fashion: (1 = 1 point, 2 and 3 = 2 points, 4 and 5 = 3 points, 6 = 4 points). The algebraic sum was calculated in each patient, where a higher total score indicates a better quality of life, with a maximal result of 120 points. Points were also calculated for each patient in every aforementioned domain (fatigue, emotions, AI symptoms, and miscellaneous questions). All patients responded to our questionnaire. There were only 6 missing answers in all questionnaires (2 in the first question, 1 in questions 11, 25, 26, and 30).

### 2.1. Ethics

The Bioethics Committee of Wroclaw Medical University approved the protocol of the study (No. 115/2021). All participants gave their oral and written consent to participate in the study in accordance with the Declaration of Helsinki.

### 2.2. Statistical Analysis

The data were analyzed with use of Statistica software for Windows (version 13.3, StatSoft, Krakow, Poland). The variables were presented as the mean with standard deviation (SD), median, and interquartile ranges (IQR). The homogeneity of the variances was determined by Levene’s test. The Shapiro–Wilk test and histograms were used to assess data distribution. Student’s *t*-test or Mann–Whitney test were performed to compare the quantitative variables. Correlations between parameters were calculated using Pearson’s test or Spearman’s rank correlation test. A *p*-value of <0.05 was considered statistically significant. The value of Cronbach’s alpha was calculated to measure scale reliability.

## 3. Results

The clinical characteristics of the PAI group are shown in Table 2. We included 32 patients (see flowchart): 20 females (62.5%) with a mean age of 50.9 ± 13.2 years, and 12 males (37.5%) with a mean age of 49.8 ± 11.4 years. Age did not differ significantly between the sexes. Males had a significantly higher body mass and BMI compared to those of women. The duration of the disease was similar in females and males. No statistically significant differences in doses of hydrocortisone or fludrocortisone between the genders were found. UFC excretion was significantly higher and DHEAS serum concentrations significantly lower in females compared to those in males (Table 2).

The detailed AddiQoL score evaluation is presented in Table 3. Cronbach’s alpha was 0.946. The total mean score was 85.0 ± 11.8, the median was 84. The total AddiQoL score was significantly higher in men than in women, indicating a better HRQoL (*p* = 0.011). Furthermore, men reached significantly higher scores in each of the four subscales (fatigue, emotional sphere, AI symptoms, and miscellaneous questions), suggesting a better HRQoL.

No significant differences in total AddiQoL, fatigue, emotional sphere, AI symptoms, and miscellaneous scores were shown between the patients with and without diabetes. Similarly, no statistically significant differences were found in total AddiQoL and the emotional sphere, AI symptoms, and miscellaneous questions scores between the patients with and without autoimmune thyroiditis. Patients with autoimmune thyroiditis had lower fatigue scores compared to those of patients without autoimmune thyroiditis (20.5 vs. 24.0, *p* = 0.034).

There was no significant difference in total AddiQoL and subscales scores when the groups were analyzed on the basis of hydrocortisone doses (patients were divided according to the mean daily dose—26.2 mg, as well as a dose of 25 mg).

In the whole study group, total AddiQoL and fatigue scores correlated negatively with age at diagnosis (r = (−)0.4084, *p* = 0.038; r = (−)0.4728, *p* = 0.015, respectively). There was a positive correlation between the emotional sphere score and body mass (r = 0.4127, *p* = 0.0365). There were statistically significant negative correlations between gonadotropins and AddiQoL subscales (LH and fatigue r = (−)0.384, *p* = 0.03; LH and emotional sphere r = (−)0.37, *p* = 0.037; FSH and fatigue r = (−)0.38, *p* = 0.032; FSH and emotional sphere r = (−)0.416, *p* = 0.018). We did not find statistically significant correlations between total AddiQoL, fatigue, emotional sphere, AI symptoms, and miscellaneous questions scores and age, BMI, duration of the disease, doses of hydrocortisone or fludrocortisone, UFC, ACTH, renin, TSH, and fT4.

We have also analyzed the correlations between testosterone, estradiol, DHEAS, gonadotropins, BMI, weight, and AddiQoL separately in males and females. In females, only the correlation between BMI and the AddiQol subscale AI symptoms (r = 0.451, *p* = 0.046) was significant. In males, there was a negative correlation between FSH and emotional the sphere and miscellaneous questions (r = (−)0.693, *p* = 0.012 and r = (−)0.577, *p* = 0.05, respectively).

## 4. Discussion

This study presents the results of an AddiQoL assessment in a group of Polish patients with PAI. To the authors’ best knowledge, this is the first published study in Poland assessing the HRQoL using this specific psychometric tool in Addison’s disease. This questionnaire was developed and validated on a group of about 600 patients, consisting also of a Polish cohort [10,11]. Previously in Poland, a group of 15 PAI patients was evaluated by Warmuz-Stangierska et al., showing increased levels of anxiety and fear with a tendency to overreact to stimuli, decreased mobile activity, and moderate to severe depression with the use of different psychological scales, not specific for Addison’s disease (Beck Depression Inventory, State-Trait Anxiety Inventory for Adults, and others) [23].

Many previous studies reported reduced QoL in patients with Addison’s disease when compared to that of healthy subjects. In a study on 54 PAI patients and 54 controls, Tiemensma et al. showed that patients suffered from more psychological morbidity with irritability, somatic arousal, and lower QoL, but there were no differences regarding personality traits [4]. Kluger et al. reported that usual activities, depression, distress, vitality, and sexual activity were the most impaired dimensions of QoL compared with those of the general population [8]. Other studies have also shown a significantly reduced subjective health status in patients with AI, a higher percentage of patients being out of work, and lower physical activity [18]. In a recent cross-over study from Norway, 494 patients with PAI presented lower QoL scores in comparison to those from normative data, especially in the domain of physical role in all age groups. Other QoL aspects like social functioning, vitality, and general health were also impaired, especially in younger age groups [9].

The specific AddiQoL questionnaire was developed to address disease-specific issues relevant to PAI in a more sensitive way than with generic questionnaires [11]. In this study, the median total AddiQoL score was 84.0 (from maximal 120 as the best result), which was slightly higher but comparable to that from other reports [14,16]. Regarding specific AddiQoL subscales, the worst result was observed in the categories of miscellaneous questions (which concerned sleep quality, sex life, susceptibility to other diseases, and recovery after diseases) and fatigue, while the emotional category was slightly better, and the least impaired was the category concerning AI symptoms. Although it cannot be definitively compared, the findings are in accordance with other results, where, with use of different HRQoL assessment tools, fatigue and the emotional sphere were affected the most [8,24]. It can be assumed that the highest scores regarding AI symptoms resulted from well-controlled disease of the recruited patients, therefore, this aspect of HRQoL was impaired the least in this study group.

In this study, there was a significant association between the female gender and impaired HRQoL. It was observed in the total AddiQoL score as well as in each of the four subcategories (fatigue, emotional sphere, AI symptoms, and miscellaneous questions). This finding is in accordance with most studies in this subject from different countries [8,9,17,24], suggesting that this phenomenon is also present in the Polish population. Interestingly, in the group of 494 patients from Norway, there were gender-specific differences, because males reported the greatest impairment in social functioning, vitality, and physical role. In females, physical role was mostly affected along with social functioning, vitality, physical function, general health, mental health, and emotional role [9]. However, other authors reported no differences in HRQoL according to gender [17]. Studies have established that in other chronic diseases, females score worse in HRQoL than males do. Therefore, it could be suggested that worse levels of HRQoL in females with PAI can be explained by a general gender difference when self-assessing well-being. In this study group, there were no differences in age at diagnosis, disease duration, or presence of other diseases that could explain impairment of HRQoL in females in comparison to that in males. However, females had significantly lower levels of DHEAS in their blood than males did. This suggests that the lack of androgens in females due to adrenal insufficiency may be partially responsible for lower HRQoL. On the other hand, data concerning androgen supplementation in PAI are inconsistent. In an initial study, premenopausal women with adrenal insufficiency who received 50 mg of DHEAS vs. placebo reported improvement in general well-being, sexual satisfaction and interest, and a decrease in depression and anxiety after 4 months of observation [20]. A crossover study showed improvement in self-esteem and fatigue but no changes in cognitive or sexual function after 3 months [25]. Other reports revealed side effects of DHEAS supplementation with no effect on fatigue, cognition or sexual function during a longer observation [6,7]. Generally, it is believed that there are some benefits in quality of life and mood but not when it comes to anxiety or sexual function in women with PAI [21]. However, these data are based on studies with small study group sizes (ranging from 19 to 32) and using different tools to measure QoL (none of them used AddiQoL). Further studies with a higher number of subjects and the use of AddiQoL are needed. Furthermore, an important aspect worthy of further analysis is a deeper understanding from the patient’s perspective. The perception of the disease and its impact on the individual’s life may have a significant impact on the quality of life.

No differences in AddiQoL scores between patients on higher vs. lower doses of hydrocortisone replacement therapy were found. UFC was higher in females in comparison to that in males, but the daily doses of hydrocortisone did not differ significantly according to gender and there was also no association between AddiQoL and UFC. Therefore, based on these data, it was not possible to draw any relevant conclusion. A point of note should be made that females who had a lower BMI and received supplementation doses similar to those of males had a higher UFC level as an indirect marker of daily hydrocortisone and presented lower HRQoL. Generally, it remains impossible to assess whether a lower HRQoL is related to glucocorticoid overdosing or rather that patients who receive higher doses have a greater disease burden and therefore need higher dosing. This study group received rather high doses of hydrocortisone (mean of 26.2 mg) but presented with neither clinical nor biochemical signs of overdosing. It is possible that the study group was too small or replacement therapy too homogenous to detect such differences. While some studies reported worse HRQoL in patients receiving higher daily hydrocortisone doses [4], others did not find such a correlation [9,17]. Moreover, there has been no association between glucocorticoid type (hydrocortisone vs. prednisolone) nor therapy regimen (twice daily vs. thrice daily) with HRQoL [8,18]. Some studies suggested that dual-release hydrocortisone preparations have an advantageous effect on HRQoL in comparison to that of standard therapy [14], while others reported no significant differences [13]. Continuous subcutaneous hydrocortisone infusion, which more closely replicates physiological cortisol release than standard oral therapy does, has led to a HRQoL improvement in some studies [12], while in others, no effect was observed [19].

This study found a negative correlation between gonadotropin concentration and AddiQoL scores. This suggests that menopause is another factor that impairs HRQoL in patients with PAI. It is important in everyday practice to not overlook this issue and possibly implement sex-steroid replacement therapy in females with PAI, whose well-being is impaired due to chronic disease and who are at risk of autoimmune premature ovarian failure.

There was no difference in AddiQoL scores between patients with and without other autoimmune diseases (diabetes and autoimmune thyroiditis) in this study group, with the exception of fatigue correlating with the presence of autoimmune thyroiditis. Other studies found that the more autoimmune comorbidities re present, the worse the HRQoL scores are [9]. Celiac disease, primary ovarian failure, and atrophic gastritis are predictors of lower AddiQoL scores [17]. It is possible that the number of patients in this study was too small to observe significant differences; however, other studies have reported an impairment of HRQoL in PAI irrespective of the presence of other endocrine and non-endocrine diseases.

In this study, total AddiQoL and fatigue scores correlated negatively with age at diagnosis, in accordance with the results of Meyer et al. [17]. In a Norwegian cohort, patients with PAI diagnosed less than 5 years before the study had a lower mental component of QoL scores in generic questionnaires than patients with a duration of the disease for over 20 years did [9]. Together with the results of this study, it may suggest that younger age at the time of diagnosis allows for better adaptation to the disease and strengthens coping strategies, which in turn affect the perceived quality of life.

Our study has some limitations. The most important one is the number of included patients, which is mostly the result of the rarity of the disease as well as the limited time of the patient enrollment phase. It was also a single-center study, which may have caused some bias. Additionally, the impact of other conventional glucocorticoid treatment regimens (e.g., dual-release) could not be included due to the lack of availability of such preparations in Poland. The use of AddiQoL, which is a specific questionnaire designed for PAI, is both a limitation and a strength of this study. The AddiQoL is sensitive enough to evaluate the HRQoL in PAI because it is adjusted to the typical symptomatology and the course of the disease and enables a more personalized approach to the patient. A high value of Cronbach’s alpha indicates internal consistency of the scale. On the other hand, it is not possible to compare the AddiQoL results with those of a healthy population. Another strength of this study is the complexity of the clinical and hormonal assessment and the autoimmune cause of PAI in all patients. However, HRQoL is not a stable parameter and may change in the course of the disease and due to other psychosocial factors, therefore, longitudinal data are needed in order to assess whether different interventions in PAI may prove some benefit in HRQoL. It is also important to take a more holistic approach in relation to the biopsychosocial model. Thus, it is crucial to take into account the quality of life and the patient’s psychological resources and limitations, as well as aspects of social health, which seem to be of great importance in chronic somatic diseases. Therefore, there is a need for further multicenter studies with a higher number of participants that would investigate the predictors of HRQoL in PAI, especially during a longitudinal observation. Additionally, to assess the impact of androgen supplementation on HRQoL, intervention studies are needed with larger groups of participants, because so far, only inconsistent data are reported in this area.

Our study brings some practical suggestions for physicians, especially in terms of the importance of estradiol replacement therapy in menopausal females of older age at PAI diagnosis, because in such patients, the HRQoL might be particularly impaired.

In conclusion, in this prospective observational study comprising 32 patients with PAI, HRQoL was assessed with a specific questionnaire (AddiQoL) and was significantly worse in females than in males. HRQoL impairment was associated with higher levels of gonadotropins and younger age at diagnosis. There was no association between HRQoL and hormonal replacement therapy regimen. This subject needs further studies, especially with a longer follow-up.

## Figures and Tables

**Table 1:** Hormonal examinations and their reference ranges.

Hormonal Examination	Reference Ranges in Females	Reference Ranges in Males
ACTH (pg/mL)	<46	<46
LH (mIU/mL)	follicular phase: 1.1–11.6 ovulation: 17.0–77.0 luteal phase: 0.7–14.7 postmenopausal: 11.3–39.8	0.8–7.6
FSH (mIU/mL)	follicular phase 2.8–11.3 ovulation 5.8–21.0 luteal phase 1.2–9.0 postmenopausal women 21.3–153.0	0.7–11.1
Estradiol (pg/mL)	follicular phase: <160.0 ovulation: 34–400.0 luteal phase: 27.0–246.0 postmenopausal women: <30.0	<56.0
Testosterone (ng/mL)	premenopausal: 0.2–0.72 postmenopausal: 0.2–0.43	<50 years: 0.72–8.53 >50 years 1.29–7.67
DHEAS (µg/dL)	35.0–430.0	80.0–560.0
fT4 (pmol/L)	9–21	9–21
TSH (IU/mL)	0.4–4	0.4–4

ACTH—adrenocorticotropic hormone; DHEAS—dehydroepiandrosterone sulfate; FSH—follicle-stimulating hormone; fT4—free thyroxine; LH—luteinizing hormone; TSH—thyroid-stimulating hormone.

**Table 2:** The clinical characteristics of the patients with primary adrenal insufficiency (women vs. men).

Group	AI			Women (*n* = 20)			Men (*n* = 12)			*p*-Value
	Mean ± SD	Median	IQR	Mean ± SD	Median	IQR	Mean ± SD	Median	IQR	
Age (years)	50.5 ± 12.4	50.0	38.5–59.5	50.9 ± 13.2	50.0	37.0–60.5	49.8 ± 11.4	48.5	40.0–58.5	0.804 *
Body mass (kg)	73.6 ± 15.7	72.5	60.0 ± 84.0	65.6 ± 9.9	64.0	58.5 ± 72.5	87.0 ± 14.7	88.5	77.0–97.0	<0.001 *
BMI (kg/m^2^)	25.8 ± 4.2	26.4	22.1–29.0	24.5 ± 4.0	24.1	20.7–27.3	28.1 ± 3.7	28.5	26.6–29.5	0.015 *
Duration of the disease (years)	12.8 ± 13.0	6.0	3.0–26.0	9.97 ± 10.2	5.0	2.0–18.5	17.4 ± 16.2	9.0	4.5–31.0	0.133 **
Age at diagnosis	37.8 ± 12.3	36.5	29–43.5	40.9 ± 13.2	38.0	34.5–49.0	32.5 ± 8.6	31.0	27.0–39.0	0.060 *
Daily dose of hydrocortisone (mg)	26.2 ± 6.0	30.0	20.0–30.0	25.6 ± 6.6	27.5	20.0–30.0	27.1 ± 5.0	30.0	22.5 ± 30.0	0.508 **
Daily dose of fludrocortisone (mg)	0.07 ± 0.05	0.05	0.03–0.1	0.06 ± 0.06	0.05	0.03–0.09	0.08 ± 0.03	0.09	0.05–0.1	0.096 **
ACTH (pg/mL)	541.9 ± 473.2	401.0	125.9–1063.5	534.8 ± 461.7	464.5	122.3–930.5	553.9 ± 512.5	301.5	161.9–1217.0	0.891 **
Urinary free cortisol (µg/day)	89.5 ± 62.5	72.8	39.8–125.5	107.4 ± 64.2	105.0	44.1–127.0	61.9 ± 50.8	55.8	28.5–64.0	0.048 **
DHEAS (µmol/L)	25.0 ± 18.5	15.0	15.0–29.0	15.0 ± 0.0	15.0	15.0–15.0	40.9 ± 22.1	40.6	24.1–52.1	<0.001 **

AI—the patients with primary adrenal insufficiency; SD—standard deviations; IQR—interquartile range; * Student’s *t*-test; ** Mann–Whitney test.

**Table 3:** Total results and subscales of the quality of life questionnaire in Addison’s disease (women vs. men).

	AI			Women (*n* = 20)			Men (*n* = 12)			*p*-Value
Group of Questions	Mean ± SD	Median	IQR	Mean ± SD	Median	IQR	Mean ± SD	Median	IQR	
Total	85.0 ± 11.8	84.0	76.5–95.5	81.1 ±8.2	79.0	76.0–85.5	91.7 ± 14.0	94.5	82.0–103.0	0.011 *
Fatigue	21.6 ± 4.1	22.0	18.5–24.5	20.3 ± 3.3	20.0	17.5–22.5	23.8 ± 4.4	24.5	20.5 ± 27.5	0.013 *
Emotional	23.2 ± 3.4	23.5	20.5–26.0	22.3 ± 3.0	22.5	19.5–24.0	24.7 ± 3.4	26.0	23.0–27.0	0.048 *
AI symptoms	26.9 ± 3.7	26.5	24.5–29.5	25.9 ± 3.0	26.0	24.0–28.0	28.6 ± 4.2	29.5	25.5–31.0	0.039 *
Miscellaneous questions	13.4 ± 2.5	13.0	11.0–15.0	12.7± 1.8	12.5	11.0–14.5	14.6 ± 3.0	14.5	12.5–17.5	0.034 *

AI—patients with primary adrenal insufficiency; SD—standard deviations; IQR—interquartile range; * Student’s *t*-test.

## Data Availability

The data presented in this study are available upon request from the corresponding author.

## References

[1] Husebye E.S., Pearce S.H., Krone N.P., Kämpe O. (2021). Adrenal insufficiency. Lancet.

[2] Frommer L., Kahaly G.J. (2019). Autoimmune Polyendocrinopathy. J. Clin. Endocrinol. Metab..

[3] Bornstein S.R., Allolio B., Arlt W., Barthel A., Don-Wauchope A., Hammer G.D., Husebye E.S., Merke D.P., Murad M.H., Stratakis C.A. (2016). Diagnosis and Treatment of Primary Adrenal Insufficiency: An Endocrine Society Clinical Practice Guideline. J. Clin. Endocrinol. Metab..

[4] Tiemensma J., Andela C.D., Kaptein A.A., Romijn J.A., van der Mast R.C., Biermasz N.R., Pereira A.M. (2014). Psychological morbidity and impaired quality of life in patients with stable treatment for primary adrenal insufficiency: Cross-sectional study and review of the literature. Eur. J. Endocrinol..

[5] Ho W., Druce M. (2018). Quality of life in patients with adrenal disease: A systematic review. Clin. Endocrinol..

[6] Løvås K., Gebre-Medhin G., Trovik T.S., Fougner K.J., Uhlving S., Nedrebø B.G., Myking O.L., Kämpe O., Husebye E.S. (2003). Replacement of dehydroepiandrosterone in adrenal failure: No benefit for subjective health status and sexuality in a 9-month, randomized, parallel group clinical trial. J. Clin. Endocrinol. Metab..

[7] Gurnell E.M., Hunt P.J., Curran S.E., Conway C.L., Pullenayegum E.M., Huppert F.A., Compston J.E., Herbert J., Chatterjee V.K.K. (2008). Long-term DHEA replacement in primary adrenal insufficiency: A randomized, controlled trial. J. Clin. Endocrinol. Metab..

[8] Kluger N., Matikainen N., Sintonen H., Ranki A., Roine R.P., Schalin-Jäntti C. (2014). Impaired health-related quality of life in Addison’s disease—Impact of replacement therapy, comorbidities and socio-economic factors. Clin. Endocrinol..

[9] Didriksen N.M., Sævik Å.B., Sortland L.S., Øksnes M., Husebye E.S. (2021). Sex-Specific Limitations in Physical Health in Primary Adrenal Insufficiency. Front. Endocrinol..

[10] Løvås K., Curran S., Øksnes M., Husebye E.S., Huppert F.A., Chatterjee V.K.K. (2010). Development of a Disease-Specific Quality of Life Questionnaire in Addison’s Disease. J. Clin. Endocrinol. Metab..

[11] Øksnes M., Bensing S., Hulting A.L., Kämpe O., Hackemann A., Meyer G., Badenhoop K., Betterle C., Parolo A., Giordano R. (2012). Quality of life in European patients with Addison’s disease: Validity of the disease-specific questionnaire AddiQoL. J. Clin. Endocrinol. Metab..

[12] Øksnes M., Björnsdottir S., Isaksson M., Methlie P., Carlsen S., Nilsen R.M., Broman J.-E., Triebner K., Kämpe O., Hulting A.-L. (2014). Continuous subcutaneous hydrocortisone infusion versus oral hydrocortisone replacement for treatment of Addison’s disease: A randomized clinical trial. J. Clin. Endocrinol. Metab..

[13] Quinkler M., Miodini Nilsen R., Zopf K., Ventz M., Øksnes M. (2015). Modified-release hydrocortisone decreases BMI and HbA1c in patients with primary and secondary adrenal insufficiency. Eur. J. Endocrinol..

[14] Giordano R., Guaraldi F., Marinazzo E., Fumarola F., Rampino A., Berardelli R., Karamouzis I., Lucchiari M., Manetta T., Mengozzi G. (2016). Improvement of anthropometric and metabolic parameters, and quality of life following treatment with dual-release hydrocortisone in patients with Addison’s disease. Endocrine.

[15] Mongioì L.M., Condorelli R.A., La Vignera S., Calogero A.E. (2018). Dual-release hydrocortisone treatment: Glycometabolic profile and health-related quality of life. Endocr. Connect..

[16] Meyer G., Koch M., Herrmann E., Bojunga J., Badenhoop K. (2018). Longitudinal AddiQoL scores may identify higher risk for adrenal crises in Addison’s disease. Endocrine.

[17] Meyer G., Hackemann A., Penna-Martinez M., Badenhoop K. (2013). What affects the quality of life in autoimmune Addison’s disease?. Horm. Metab. Res..

[18] Bleicken B., Hahner S., Loeffler M., Ventz M., Decker O., Allolio B., Quinkler M. (2010). Influence of hydrocortisone dosage scheme on health-related quality of life in patients with adrenal insufficiency. Clin. Endocrinol..

[19] Gagliardi L., Nenke M.A., Thynne T.R.J., von der Borch J., Rankin W.A., Henley D.E., Sorbello J., Inder W.J., Torpy D.J. (2014). Continuous subcutaneous hydrocortisone infusion therapy in Addison’s disease: A randomized, placebo-controlled clinical trial. J. Clin. Endocrinol. Metab..

[20] Arlt W., Callies F., van Vlijmen J.C., Koehler I., Reincke M., Bidlingmaier M., Huebler D., Oettel M., Ernst M., Schulte H.M. (1999). Dehydroepiandrosterone replacement in women with adrenal insufficiency. N. Engl. J. Med..

[21] Wierman M.E., Kiseljak-Vassiliades K. (2022). Should Dehydroepiandrosterone Be Administered to Women?. J. Clin. Endocrinol. Metab..

[22] Espiard S., McQueen J., Sherlock M., Ragnarsson O., Bergthorsdottir R., Burman P., Dahlqvist P., Ekman B., Engström B.E., Skrtic S. (2021). Improved Urinary Cortisol Metabolome in Addison Disease: A Prospective Trial of Dual-Release Hydrocortisone. J. Clin. Endocrinol. Metab..

[23] Warmuz-Stangierska I., Baszko-Błaszyk D., Sowiński J. (2010). Emotions and features of temperament in patients with Addison’s disease. Endokrynol. Pol..

[24] Løvås K., Loge J.H., Husebye E.S. (2002). Subjective health status in Norwegian patients with Addison’s disease. Clin. Endocrinol..

[25] Hunt P.J., Gurnell E.M., Huppert F.A., Richards C., Prevost A.T., Wass J.A., Herbert J., Chatterjee V.K.K. (2000). Improvement in mood and fatigue after dehydroepiandrosterone replacement in Addison’s disease in a randomized, double blind trial. J. Clin. Endocrinol. Metab..

